# Beyond the Gut: Extra-Enteric Digestive Manifestations of Inflammatory Bowel Disease—A Personalized Medicine Perspective and Comprehensive Review

**DOI:** 10.3390/jpm16040219

**Published:** 2026-04-16

**Authors:** Maria Rogalidou, Maria-Veatriki Christodoulou, Alexandros Skamnelos, Dimitrios K. Christodoulou

**Affiliations:** 1 Division of Gastroenterology Hepatology & Nutrition, 1st Pediatrics Department, National and Kapodistrian University of Athens, “Agia Sofia” Children’s Hospital, Thivon & Papadiamantopoulou Street, 11527 Athens, Greece; 2 Radiology Department, University Hospital of Ioannina, 45500 Ioannina, Greece; 3 Division of Gastroenterology, University Hospital of Ioannina, 45500 Ioannina, Greece; 4 Division of Gastroenterology, Medical School of Ioannina, 45500 Ioannina, Greece

**Keywords:** inflammatory bowel disease (IBD), hepatobiliary involvement, pancreatic manifestations, splenic involvement, oral manifestations, extraintestinal digestive manifestations of IBD, Crohn’s disease, ulcerative colitis

## Abstract

Inflammatory bowel disease (IBD)—including Crohn’s disease, ulcerative colitis, and indeterminate colitis—is a chronic immune-mediated condition that primarily affects the intestinal mucosa but often presents with extraintestinal digestive manifestations, which are important yet frequently underrecognized sources of morbidity. These heterogeneous manifestations reflect diverse genetic, microbial, immunologic, and environmental influences, highlighting the value of a personalized medicine approach. Hepatobiliary involvement affects IBD adults patients and is even more common in children, ranging from mild liver enzyme elevations to severe complications such as liver failure, with autoimmune disorders, cholelithiasis, portal vein thrombosis, and non-alcoholic fatty liver disease as key considerations. Pancreatic manifestations may include autoimmune or acute pancreatitis, often linked to gallstones, thiopurine exposure, or duodenal Crohn’s disease, while splenic abnormalities, such as granulomatous lesions, splenomegaly, or functional hyposplenism, reflect systemic immune dysregulation. Oral findings—including aphthous ulcers, periodontitis, pyostomatitis vegetans, and granulomatous cheilitis—can serve as early, patient-specific indicators of disease activity. Personalized approaches, encompassing investigations tailored to the individual profile and selected targeted therapies, are essential for improving diagnostic accuracy, preventing complications, and optimizing multidisciplinary care in patients with IBD.

## 1. Introduction

Inflammatory bowel disease (IBD), which includes Crohn’s disease (CD), ulcerative colitis (UC), and indeterminate colitis (IC), is a chronic inflammatory disorder of the gastrointestinal tract resulting from interactions between genetic susceptibility, immune dysregulation, and environmental triggers [1,2]. While IBD primarily affects the intestine, it is increasingly recognized as a systemic disease with extraintestinal manifestations (EIM)—inflammatory conditions occurring outside the gut [3,4]. EIM are common, contribute substantially to morbidity and mortality [5,6], and often require a personalized approach for diagnosis and management.

EIM are distinct from IBD-related complications, which result directly from intestinal inflammation rather than representing independent inflammatory processes. They are believed to arise from the same pathogenic mechanisms driving intestinal inflammation but occur at extraintestinal sites [2]. Mechanistically, EIM are classified as specific, reactive, associated, or treatment-induced [4,7]. Specific EIM represent the same disease process as IBD outside the gut; reactive EIM share pathogenic pathways without identical histology; associated EIM occur more frequently in IBD patients, though the underlying link remains unclear; and treatment-induced EIM develop during therapy and typically resolve after cessation [3]. It remains uncertain whether EIM result directly from intestinal inflammation or from shared genetic and immune predispositions.

Among hepatobiliary complications, fatty liver is the most common, whereas primary sclerosing cholangitis (PSC) is the most specific to IBD. Less common disorders include autoimmune hepatitis–PSC overlap syndrome, primary biliary cholangitis, hepatic amyloidosis, granulomatous hepatitis, cholelithiasis, portal vein thrombosis, and liver abscess [8]. The type and prevalence of these manifestations vary according to the underlying IBD subtype. Some IBD treatments may also induce liver toxicity, although serious adverse events are rare [5].

Pancreatic manifestations occur more frequently in IBD patients than in the general population. These range from asymptomatic laboratory or imaging abnormalities, including steatosis, to clinically significant conditions such as acute and chronic pancreatitis, autoimmune pancreatitis, and exocrine pancreatic insufficiency, which may arise from IBD itself or its treatments [9].

Splenic abnormalities in IBD patients may result from conditions such as portal hypertension, myeloproliferative disorders, amyloidosis, or infectious and granulomatous involvement. In CD, the spleen is often enlarged, which is associated with worse clinical outcomes and may regress with treatment. In contrast, patients with UC usually have normal or smaller spleens [10].

Oral lesions are more common in CD than in UC. CD-specific findings include mucosal tags, cobblestoning, and deep linear ulcerations, whereas pyostomatitis vegetans is more specific to UC. Most oral manifestations appear independent of intestinal disease activity, although further studies are needed to clarify their prevalence and clinical relevance [11].

This review summarizes digestive extraintestinal manifestations (DEIMs), focusing on epidemiology, genetics, and pathogenesis, and highlights the personalized approach needed for the investigation and management of these patients.

## 2. Definition

Extraintestinal manifestations (EIMs) in IBD refer to inflammatory conditions that occur outside the gastrointestinal tract. Their pathogenesis may result from the extension or translocation of immune responses from the intestine, or they may represent independent inflammatory events that are either perpetuated by IBD or share a common genetic or environmental predisposition with the disease [12].

## 3. Pathogenesis

Digestive extraintestinal manifestations (DEIMs) in IBD typically arise from a shared network of pathogenic mechanisms. Central to their development is immune dysregulation, in which T- and B-cell–mediated responses—sometimes triggered by antigens common to both the gut and extraintestinal tissues—target distant organs. Increased intestinal permeability (“leaky gut”) allows microbial products and inflammatory cytokines to enter the systemic circulation, promoting tissue injury in the liver, pancreas, spleen, and oral mucosa. Genetic predisposition, including HLA and other immune-regulatory variants, further modulates susceptibility, while environmental and patient-specific factors such as medications, microbial composition, and nutritional status shape the individual pattern of manifestations [3,12,13].

**Figure 1:**
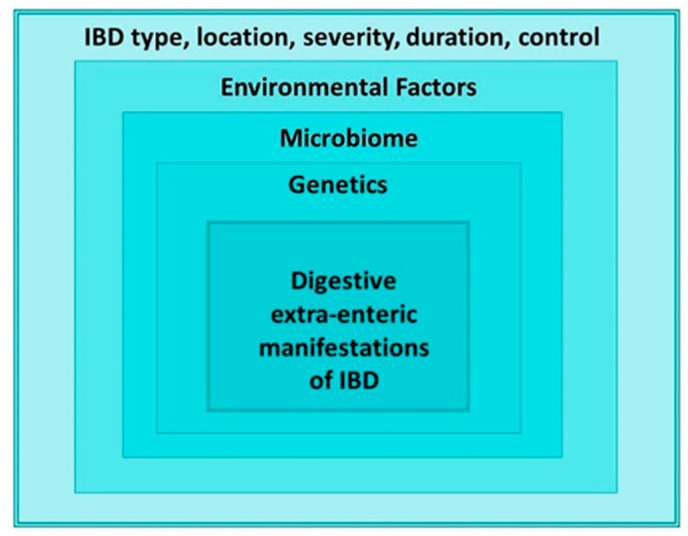
The main pathogenetic mechanisms of Digestive extra intestinal manifestations of IBD.

The main pathogenetic mechanisms are summarized in Figure 1.

Recognizing this personalized interplay between gut inflammation and systemic effects is essential for tailoring monitoring and therapy, addressing not only intestinal disease but the full spectrum of organ involvement.

### 3.1. Genetic Factors

Genetic factors play a central role in the development of DEIMs in IBD, particularly hepatobiliary involvement. Variants in the HLA region, strongly associated with PSC—such as HLA-DRB10301 (DR3), HLA-B8, and HLA-DRB30101 (DRw52a) [14]—influence antigen presentation and T-cell activation, reflecting a shared gut–liver immune axis [9,10,12,13,14,15,16]. Beyond HLA, genome-wide studies have identified multiple immune-regulatory loci affecting IL-23/Th17 signaling, TNF pathways, epithelial barrier function, and host–microbiome interactions, which contribute to both intestinal and extraintestinal inflammation [9,10,12,13,14,15,16].

In PSC-IBD, additional non-HLA loci define a distinct genetic profile, suggesting that DEIMs arise from a genetically primed systemic immune environment targeting specific organs [9,10,12,13,14,15,16]. Pancreatic manifestations—particularly type 2 autoimmune pancreatitis (AIP) linked to UC—also reflect overlapping gut–pancreas immune pathways, with HLA class II alleles (e.g., DRB10405–DQB10401) and other immune-related variants implicating dysregulated antigen presentation and adaptive immunity [17,18].

Splenic involvement, including reactive splenomegaly or hyposplenism, appears to result from systemic immune activation and shared cytokine or lymphoid trafficking influenced by IBD risk alleles [15,16]. Moreover, genes such as IL23R, NOD2, and ATG16L1 modulate mucosal immunity, epithelial barrier integrity, and host–microbiome interactions; in combination with HLA variants and Th17/TNF signaling, they create a systemic immune milieu capable of extending intestinal inflammation to the oral cavity and other digestive organs [15,16,19].

Collectively, these findings indicate that DEIMs in IBD reflect a genetically primed systemic immune profile, in which organ-specific inflammation arises from shared immune pathways linking the gut with other digestive tissues [3,9,15,16,19].

### 3.2. Enviromental Factors

Environmental factors play a critical role in the development and severity of DEIMs in IBD, affecting the hepatobiliary system, pancreas, gallbladder, spleen, and oral cavity. Smoking, Westernized diets, air pollution, and emerging environmental pollutants have been linked to immune dysregulation, impaired barrier function, and alterations in gut microbiota, contributing to both intestinal inflammation and systemic organ involvement, including primary sclerosing cholangitis, autoimmune hepatitis, non-alcoholic fatty liver disease, pancreatitis, gallstones, and oral lesions such as aphthous ulcers [1,20,21,22].

Medication exposures (e.g., thiopurines, mesalamine, corticosteroids), alcohol consumption, and nutrient deficiencies (vitamin D, iron, B12) further modulate these risks by promoting hepatotoxicity, pancreatic injury, or mucosal susceptibility [1,20]. Collectively, these environmental exposures interact with host genetics and immune pathways, emphasizing the importance of preventative strategies that target modifiable risk factors to mitigate DEIMs in IBD [20,21].

### 3.3. The Role of Microbiome

The gut microbiota appears to influence the development and phenotypic expression of DEIMs in IBD through multiple mechanistic pathways. Patients with IBD who develop extraintestinal manifestations exhibit distinct fecal microbiota profiles compared with those without such manifestations, including reduced microbial diversity and altered abundances of taxa associated with immune-mediated disease and barrier integrity. These observations suggest that dysbiosis may contribute to systemic immune activation and extraintestinal targeting [1,23].

Dysbiosis is particularly notable in PSC–IBD, with unique microbial signatures and shifts in bile acid metabolism that may interact with mucosal immunity and the gut–liver axis to promote cholangiopathy [23,24]. Mechanistic models propose that microbial translocation across a compromised epithelial barrier, microbial metabolite signaling (e.g., bile acids, short-chain fatty acids), and molecular mimicry can drive aberrant immune cell trafficking and inflammation in organs such as the liver, pancreas, spleen, and oral mucosa [25].

These findings highlight the microbiome’s role not only in intestinal disease but also in shaping the systemic inflammatory milieu underlying DEIMs, supporting the exploration of microbiota-targeted strategies to mitigate extraintestinal involvement.

### 3.4. IBD Characteristics and DEIM Risk

The development and severity of DEIMs in IBD are influenced by disease phenotype, inflammatory burden, anatomical location, and disease duration. Extensive UC and colonic-predominant CD are strongly associated with hepatobiliary complications, likely due to sustained portal cytokine exposure and gut–liver axis activation. Gallstones are more prevalent in IBD patients, particularly in those with CD, but not in UC [26]. Ileocolonic CD and active intestinal inflammation increase the risk of pancreatic involvement, including acute and chronic pancreatitis, with autoimmune pancreatitis also reported in UC; recurrence of AIP is more common in IBD patients, partly mediated by immune dysregulation and treatment exposure [1,26].

Persistent systemic inflammation and long-standing disease correlate with splenic enlargement and altered splenic immune function, reflecting chronic immune activation [2,3,27]. Oral manifestations, which often parallel intestinal activity, are more frequent in CD—typically presenting as aphthous ulcers and cobblestoning—whereas pyostomatitis vegetans is a rare but specific finding in UC, particularly in colonic and active disease phenotypes [19]. Non-alcoholic fatty liver disease (NAFLD) is slightly more prevalent in CD, especially in patients with upper GI involvement, while IBD medications appear to have minimal impact [28,29,30].

Overall, higher inflammatory burden and prolonged disease duration amplify systemic cytokine release and microbial translocation, increasing both the likelihood and severity of organ involvement. However, these associations are not deterministic; individual variability reflects the interplay of disease phenotype with genetic susceptibility, metabolic profile, microbiome composition, and therapeutic exposures [1].

## 4. Epidemiology

While the absolute prevalence of DEIMs varies by population and diagnostic criteria, hepatobiliary and pancreatic involvement affects a clinically significant proportion of patients with CD and UC. PSC is strongly associated with IBD, particularly UC: most PSC patients have coexisting UC, fewer have CD, yet only a small fraction of UC and CD patients develop PSC. In UC, extensive colonic involvement increases risk compared with left-sided disease [31]. In a recent systematic review and meta-analysis (PROSPERO) including 118 studies with 1,729,128 patients, the pooled prevalence of overall hepatic manifestations was 3.49%. NAFLD was the most common (26.1%), followed by biliary stones (4.1%) and PSC (1.67%), while autoimmune hepatitis (0.51%) and portal vein thrombosis (0.21%) were rare; substantial heterogeneity was noted across studies [32]. Pancreatic involvement is uncommon but clinically relevant. Acute pancreatitis occurs in 1–3% of patients, more often in CD than UC [33]; autoimmune pancreatitis is rare (~0.6%), chronic pancreatitis <1%, and asymptomatic enzyme elevations may be observed in up to 20% of cases [26,34]. Oral manifestations are a well-recognized EIM of IBD. In adults, lesions such as aphthous ulcers, mucosal tags, cobblestoning, cheilitis, and pyostomatitis vegetans occur in approximately 5–50% of patients [35], more frequently in CD than UC. A recent systematic review reported prevalence ranging from 0.7% to ~37% in adults and 7–23% in pediatric patients, reflecting heterogeneity in study design and lesion definitions. Overall, oral involvement is common but variable, encompassing both specific and nonspecific mucosal changes associated with IBD.

## 5. Digestive System Extraintestinal Manifestations (DEIMs)

The main DEIMs of IBD are summarized in Table 1 and include hepatobiliary, pancreatic, splenic, and oral manifestations. Hepatobiliary conditions comprise primary sclerosing cholangitis, fatty liver, autoimmune hepatitis, and cholelithiasis, while thrombotic complications such as portal vein thrombosis may occur due to the disease-associated hypercoagulable state. Pancreatic involvement includes acute pancreatitis, chronic pancreatitis, and autoimmune pancreatitis. Splenic manifestations can present as splenomegaly or functional hyposplenism, and oral manifestations encompass aphthous ulcers, cobblestoning of the oral mucosa, dental caries, and other mucosal or periodontal abnormalities. These manifestations may occur during active intestinal flares or independently of intestinal activity and often require specialized management.

**Figure 2:**
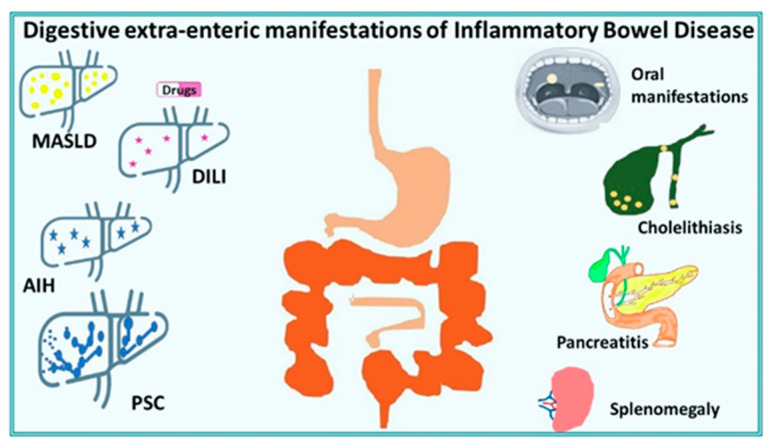
The main digestive extraintestinal manifestations of IBD.

The main DEIMs appear in Figure 2.

### 5.1. Hepatobiliary Manifestations

#### 5.1.1. Primary Sclerosing Cholangitis (PSC)

PSC is a rare, chronic cholestatic liver disease with an incidence of 0.87 per 100,000 persons/year and a prevalence of 13.53 per 100,000 [36]. Its rising incidence likely reflects improved recognition, advances in imaging, and increased survival [36,37]. PSC predominantly affects men (~2:1) and typically presents in early to middle adulthood [31,37]. PSC encompasses heterogeneous phenotypes involving intrahepatic and/or extrahepatic bile ducts. Large-duct PSC is characterized by multifocal strictures and a “beaded” appearance on MRCP or ERCP, whereas small-duct PSC (SD-PSC) presents with normal cholangiography but typical histological features, generally following a milder disease course [36,37].

PSC is strongly associated with inflammatory bowel disease (IBD), affecting approximately 70% of patients—most commonly ulcerative colitis (~60–65%) and less frequently Crohn’s disease (~5–15%) [38]. Conversely, PSC occurs in a minority of IBD patients (~2.5% in UC and ~1% in CD) [38]. SD-PSC accounts for 10–20% of PSC-IBD cases and is defined by persistent cholestatic liver enzyme elevation, normal imaging, and histological evidence of periductal fibrosis [38,39,40,41,42]. Although SD-PSC has a more favorable prognosis and lower risk of cholangiocarcinoma, 10–20% of cases progress to large-duct disease, necessitating long-term surveillance [40,41,42]. Notably, PSC-IBD is associated with an increased risk of colorectal neoplasia irrespective of intestinal disease activity [40,43].

Diagnosis relies on persistent cholestatic liver enzyme abnormalities, characteristic cholangiographic findings in large-duct disease, and exclusion of secondary causes, with liver biopsy reserved for SD-PSC or atypical cases [37]. The 2024 diagnostic criteria emphasize a multimodal approach integrating imaging, laboratory findings, IBD status, and histology, while excluding IgG4-related and secondary sclerosing cholangitis, malignancy, and other liver diseases [41]. Large-duct PSC requires characteristic imaging plus at least one supportive feature, whereas SD-PSC requires normal imaging with supportive laboratory, histological, and clinical findings [41,46].

Management remains largely supportive and surveillance-based. It includes monitoring liver disease progression, controlling IBD activity, and screening for hepatobiliary and colorectal malignancies. Endoscopic intervention is indicated for dominant strictures, while liver transplantation remains the only curative option for advanced disease [44]. No disease-modifying therapies are currently approved; ursodeoxycholic acid improves biochemical markers without clear survival benefit [45]. Emerging therapies—including FXR agonists, FGF analogues, immunomodulators, and microbiome-targeted approaches—highlight the shift toward mechanism-based and personalized treatment strategies [45].

Given the marked heterogeneity of PSC-IBD, a personalized medicine approach is essential. Hepatic and intestinal disease activity often diverge, requiring parallel monitoring using liver biochemistry and imaging alongside endoscopic and biomarker assessment of IBD [40,47,48]. Surveillance should be individualized, with annual colonoscopy recommended from the time of PSC diagnosis due to the elevated colorectal cancer risk, and advanced techniques such as chromoendoscopy improving dysplasia detection [47,48,49].

Therapeutic decision-making must account for individual risk factors, disease phenotype (small- vs. large-duct), presence of dominant strictures, and potential hepatotoxicity of IBD therapies. The timing of interventions—including ERCP and colectomy—should be guided by combined disease activity. Evaluation for liver transplantation should incorporate IBD severity, nutritional status, and comorbidities. Overall, a multidisciplinary and patient-centered, personalized strategy is critical to optimize outcomes and reflects the core principles of personalized medicine in PSC-IBD management.

#### 5.1.2. Autoimmune Hepatitis (AIH) & AIH)/PSC Overlap Syndrome

Autoimmune hepatitis (AIH)/PSC overlap syndrome is a distinct but uncommon immune-mediated hepatobiliary phenotype characterized by features of both AIH and PSC, frequently occurring in patients with IBD. AIH–PSC overlap occurs in 5–14% of adults and up to 30–40% of pediatric PSC patients, whereas PSC features are observed in 2–8% of AIH patients [50,51,52]. A recent meta-analysis of 172 studies confirmed a bidirectional association between autoimmune liver diseases (AILD) and IBD: IBD was present in 32.1% of AILD patients (62.8% PSC, 3.5% AIH, 2.0% PBC), while AILD occurred in 2.3% of IBD patients, supporting the concept of a gut–liver axis [53]. Clinically, patients present with mixed hepatocellular and cholestatic enzyme elevations, hypergammaglobulinemia (IgG), positive autoantibodies (ANA/SMA), interface hepatitis on biopsy, and cholangiographic features of PSC [50,51,52]. Diagnosis requires careful clinicopathologic correlation, as overlap syndromes are increasingly considered a spectrum rather than distinct entities [51,52].

Management primarily targets the AIH component using corticosteroids ± azathioprine, while monitoring PSC progression. Relapse is common, and advanced fibrosis or cirrhosis may necessitate liver transplantation [50,51,52]. In patients with IBD, therapy should be coordinated with optimal control of intestinal inflammation, regular hepatobiliary monitoring, and colorectal cancer surveillance, highlighting the importance of a multidisciplinary, individualized approach to optimize long-term outcomes [51,52,53].

#### 5.1.3. Cholelithiasis (Gallstones)

Patients with IBD, particularly those with CD involving the ileum or a history of ileal resection, are at increased risk of gallstones due to impaired bile acid absorption and bile stasis. A recent meta-analysis of 10 studies demonstrated that gallstones are significantly more common in IBD: UC patients had higher prevalence than controls (OR = 1.67, 95% CI: 1.32–2.11), and CD patients had an even greater risk (OR = 2.22, 95% CI: 1.82–2.69) [59]. Regional analyses confirmed elevated risk in both Asia and Europe, providing robust evidence for the association between gallstones and IBD.

A population-based cohort study further showed that CD increases the risk of all types of cholelithiasis, including gallbladder and bile duct stones (aHR 1.76–2.78), whereas UC is primarily associated with gallbladder stones (aHR 1.44) but not bile duct or intrahepatic stones [60]. Prevalence in CD ranges from 11–34%, while UC patients have a risk closer to the general population (5.5–15%) [31]. Gallstones may be asymptomatic or present with biliary colic, cholecystitis, or choledocholithiasis, and diagnosis is usually made by ultrasound.

Management is personalized: asymptomatic stones are often observed, whereas symptomatic stones require laparoscopic cholecystectomy, with ERCP indicated for bile duct stones. Optimizing IBD control is essential to reduce gallstone formation and related complications, and ursodeoxycholic acid prophylaxis may be considered in selected patients, particularly those with extensive ileal resection [31,59,60].

#### 5.1.4. Metabolic Associated Steatotic Liver Disease (MASLD)

Fatty liver disease, now termed metabolic dysfunction–associated steatotic liver disease (MASLD; formerly NAFLD), is one of the most common hepatobiliary manifestations in patients with IBD. A recent global meta-analysis of 64 studies including 1,532,811 individuals reported a pooled prevalence of 25.4% (95% CI: 23.1–27.8%), indicating that approximately one in four IBD patients is affected [30]. Prevalence was higher in adults (26.0%) than in pediatric patients (7.0%), and in males (32.1%) compared with females (22.9%). By IBD subtype, prevalence was 21.4% in UC and 22.8% in CD, with substantial geographic variation ranging from 16.0% in Asia to 32.0% in Europe [30]. Rates were higher when assessed using transient elastography or liver biopsy. Additional meta-analyses report pooled prevalence estimates between 24–34%, depending on diagnostic modality and population characteristics [28,54].

Risk factors for MASLD in IBD include obesity, insulin resistance, type 2 diabetes mellitus, dyslipidemia, corticosteroid exposure, sedentary lifestyle, increasing age, longer IBD duration, and prior bowel resection [28,30,54]. CD, particularly with small-bowel involvement, appears to confer a greater risk than UC, likely due to higher inflammatory burden, nutritional alterations, and more frequent resections [29]. These findings underscore the combined role of metabolic dysfunction and chronic intestinal inflammation via the gut–liver axis. No consistent association has been observed between MASLD and IBD medications, including 5-ASA, azathioprine, biologics, or corticosteroids [30].

Clinically, MASLD in IBD is often asymptomatic and detected incidentally through elevated liver enzymes or imaging showing hepatic steatosis. Diagnosis relies on ultrasound or cross-sectional imaging, with noninvasive fibrosis assessment (e.g., elastography or fibrosis scores) to identify advanced disease.

Management is personalized, focusing on lifestyle modification, optimization of metabolic risk factors, and careful coordination of IBD therapy to minimize hepatotoxicity. Optimizing IBD control may reduce systemic inflammation and limit hepatic progression. Patients with advanced fibrosis require hepatology referral and structured monitoring to prevent progression to cirrhosis or hepatocellular carcinoma [28,29,30,54].

#### 5.1.5. Drug-Induced Liver Injury (DILI)

Patients with IBD are frequently treated with combinations of immunomodulators, biologics, corticosteroids, 5-aminosalicylic acid (5-ASA), methotrexate, and antibiotics over prolonged periods. This polypharmacy, often spanning months to years, increases the risk of drug-induced liver injury (DILI) and complicates identification of the causative agent. The risk is further compounded by disease-related factors such as prior bowel resections, chronic inflammation, and metabolic comorbidities [55,56,57,58].

DILI is a significant hepatobiliary complication in IBD arising from multiple pharmacologic therapies. Thiopurines (azathioprine, 6-mercaptopurine) are associated with hepatotoxicity in 3–14% of patients, including transaminase elevations, cholestatic injury, or rarely, nodular regenerative hyperplasia, influenced by TPMT metabolism [55,57]. Methotrexate carries a higher hepatotoxic risk in IBD than in non-IBD populations; pooled analyses of 128,876 patients reported total liver injury in 11.2% of IBD patients (vs. 3% in non-IBD; RR = 3.76), MTX discontinuation in 3.3% (vs. 0.7%; RR = 5), and liver fibrosis in 3.1% (vs. 0.1%; RR = 38.6), highlighting the need for individualized monitoring [56]. Corticosteroids may induce hepatic steatosis and mild enzyme abnormalities, particularly with prolonged high-dose use [55]. 5-ASA compounds are generally safe, with rare idiosyncratic hepatotoxicity [55,58]. Biologics—including anti-TNF agents, anti-integrins, and anti-IL12/23 therapies—generally carry low hepatotoxic risk, though rare autoimmune-like hepatitis or idiosyncratic injury occurs (<1–2%) [55,57,58]. JAK inhibitors infrequently cause mild, transient transaminase elevations [58]. Antibiotics, such as metronidazole or ciprofloxacin, may rarely induce hepatocellular or cholestatic injury, especially with prolonged use [55,58].

Personalized management is essential. Baseline liver assessment, periodic monitoring, and therapy adjustments based on individual risk factors—including age, comorbidities, prior liver disease, IBD phenotype, and prior bowel resections—are recommended. Decisions regarding dose modification, drug switching, or prophylactic strategies should be made in collaboration with hepatology. Most hepatotoxic events resolve after drug withdrawal, although autoimmune-like reactions may require corticosteroid therapy. This approach underscores the importance of a patient-centered, multidisciplinary strategy to optimize both intestinal and hepatic outcomes in IBD [55,56,57,58].

#### 5.1.6. Budd-Chiari Syndrome or Portal Vein Thrombosis

Patients with IBD are at increased risk of thrombotic complications, including Budd-Chiari syndrome (BCS), portal vein thrombosis (PVT), and portal venous system thrombosis (PVST), due to a hypercoagulable state associated with active intestinal inflammation, hospitalization, surgery, and corticosteroid therapy [31,71,72]. Although these vascular events are relatively rare, they can lead to hepatic congestion, portal hypertension, and acute liver failure. Clinical features may include abdominal pain, hepatomegaly, ascites, or abnormal liver function tests. Diagnosis is typically established using Doppler ultrasound, CT, or MRI.

A systematic review of 36 studies including 143,659 IBD patients found that, in those without a history of colorectal surgery, PVST prevalence was 0.99% in UC, 1.45% in CD, and 0.40% in unclassified IBD. Among patients undergoing colorectal surgery, PVST incidence increased substantially to 6.95% in UC, 2.55% in CD, and 3.95% in unclassified IBD, with higher rates observed when imaging examinations were performed. Post-surgical risk factors included preoperative corticosteroid therapy (OR = 3.11; 95% CI: 1.02–9.53) and urgent surgery (OR = 1.80; 95% CI: 1.08–2.99). Mortality after PVST was 4.3% (34/789) [71].

Management is individualized and may involve anticoagulation, optimization of underlying IBD activity, and, in severe cases, interventional radiology or liver transplantation. Early recognition, risk stratification, and coordination among gastroenterology, hepatology, and surgery teams are essential to optimize outcomes [31,71,72].

### 5.2. Pancreatic Manifestations

Patients with IBD may develop a range of pancreatic disorders, including acute pancreatitis, chronic pancreatitis, autoimmune pancreatitis (AIP), and exocrine pancreatic insufficiency (EPI). These manifestations can arise from the underlying IBD, medications, or autoimmune mechanisms, and can significantly impact patient morbidity [61,62,63,64,65].

Acute pancreatitis is the most commonly reported pancreatic complication in IBD, with an estimated incidence of ~1–4%, slightly higher in CD than in UC [61,62,63]. Etiologies include medications (thiopurines such as azathioprine and 6-mercaptopurine, 5-ASA/mesalamine, corticosteroids), gallstones, hypertriglyceridemia, and IBD-related autoimmune mechanisms. Drug-induced pancreatitis is typically mild and reversible, though recurrence may occur if the offending agent is reintroduced [61,63,65].

Autoimmune pancreatitis, particularly type 2 AIP, is more frequently associated with IBD, predominantly UC. Type 2 AIP is characterized by focal pancreatic enlargement, irregular pancreatic duct narrowing, and neutrophilic infiltration on histology. Serum IgG4 may be elevated in some cases. Type 2 AIP generally responds well to corticosteroids, rarely relapses, and often parallels IBD activity, in contrast to type 1 AIP [34,61,64].

Exocrine pancreatic insufficiency may result from chronic inflammation, prior small bowel resections, or chronic pancreatitis. Clinical features include steatorrhea, weight loss, and fat-soluble vitamin deficiencies. Diagnosis relies on fecal elastase-1 measurement or direct pancreatic function tests [61,65].

Personalized management involves identifying the underlying etiology (drug-induced, autoimmune, or structural), optimizing IBD control, discontinuing offending medications if necessary, and initiating enzyme replacement therapy for EPI. Close monitoring and coordination with gastroenterology and pancreatic specialists are recommended to improve outcomes and reduce complications.

Management is tailored according to etiology:

• Drug-induced pancreatitis: discontinue the offending agent.
• Autoimmune pancreatitis: corticosteroids; immunomodulators for steroid-dependent cases.
• Exocrine pancreatic insufficiency: pancreatic enzyme replacement therapy and nutritional support.

Control of underlying IBD activity is essential, as active intestinal inflammation can exacerbate pancreatic manifestations [61,62,63,64,65]. Although relatively uncommon, pancreatic involvement should be suspected in patients presenting with abdominal pain, unexplained elevations of pancreatic enzymes, or malabsorption. Early recognition, accurate diagnosis, and individualized management are best achieved through a multidisciplinary approach involving gastroenterology, pancreatic specialists, and nutrition support teams [61,62,63,64,65].

### 5.3. Splenic Manifestations

Patients with IBD can experience a range of splenic abnormalities, from structural changes to functional and immunologic alterations. Although less frequently highlighted than hepatobiliary or pancreatic complications, splenic involvement can contribute to morbidity and influence management strategies [27,31,66].

Splenomegaly is relatively common and often reflects chronic systemic inflammation, immune activation, or portal hypertension in patients with concomitant hepatobiliary disease. Functional hyposplenism increases susceptibility to infections and impairs clearance of circulating immune complexes. Splenic granulomas, although rare and most frequently observed in CD, represent noncaseating granulomatous inflammation driven by chronic immune activation or microbial antigens. These lesions are usually asymptomatic but may occasionally cause left upper quadrant discomfort or early satiety. Imaging modalities (ultrasound, CT, MRI) can detect hypoechoic or nodular lesions, while histopathology is required to confirm granulomas and exclude infectious or neoplastic causes [27,31,66].

Personalized management depends on the type and severity of splenic involvement. Asymptomatic splenomegaly or granulomas can be monitored with periodic imaging, whereas symptomatic or enlarging lesions may require surgical intervention. Optimizing control of underlying IBD is essential, as active intestinal inflammation can exacerbate splenic abnormalities. Patients with functional hyposplenism should receive appropriate vaccinations and infection prophylaxis, emphasizing a multidisciplinary and individualized approach [27,31,66].

### 5.4. Oral Manifestations

Oral manifestations are common extraintestinal features of IBD, particularly in Crohn’s disease, and can occasionally precede intestinal symptoms. Specific oral lesions in Crohn’s disease include orofacial granulomatosis, lip swelling, cobblestoning of the buccal mucosa, linear ulcers, mucogingivitis, and mucosal tags, whereas ulcerative colitis is more often associated with pyostomatitis vegetans [19,35,68]. Non-specific manifestations, such as recurrent aphthous ulcers, angular cheilitis, glossitis, xerostomia, periodontal inflammation, and dental caries, are also frequently observed. The prevalence of oral lesions ranges from 20–50% in Crohn’s disease and 5–10% in ulcerative colitis [19,66,67,68,69]. Dental caries, commonly assessed using the DMFT index (Decayed, Missing, Filled Teeth), are more frequent in IBD patients than healthy controls, likely due to enamel defects, xerostomia, altered salivary composition, and dietary factors [70].

The pathogenesis of oral manifestations is multifactorial, reflecting shared mechanisms with intestinal disease, including immune dysregulation (Th1/Th17 pathways), chronic inflammation, genetic susceptibility, microbiome alterations, nutritional deficiencies (iron, folate, vitamin B12), and medication effects [19,35,66,69]. The oral–gut axis highlights bidirectional interactions between oral and intestinal microbiota, with periodontal inflammation potentially exacerbating systemic disease activity [68].

Personalized, multidisciplinary management is essential. Strategies include preventive dental care, nutritional counseling, management of xerostomia, regular dental monitoring, and coordinated care between gastroenterologists and dental professionals to optimize both oral and systemic health [19,35,66,68,69,70]. Early recognition and targeted treatment of oral lesions improve patient comfort and quality of life while providing insight into intestinal disease activity. Periodic oral assessment should therefore be an integral component of IBD care, emphasizing a patient-centered approach to managing both oral and systemic manifestations.

## 6. Existing Controversies and Limitations

Extraintestinal manifestations (EIMs) of IBD—including hepatobiliary, pancreatic, splenic, and oral involvement—present several controversies and evidence gaps.

In hepatobiliary disease, the clinical benefit of ursodeoxycholic acid in PSC remains debated, emerging therapies lack robust validation, and optimal surveillance strategies for small-duct PSC and PSC-IBD–related colorectal cancer are unclear. The management of AIH–PSC overlap raises questions regarding classification and best immunosuppressive strategies, while drug-induced liver injury (DILI) is complicated by polypharmacy and lacks standardized monitoring protocols.

Thrombotic complications, such as Budd-Chiari syndrome and portal vein thrombosis, are rare, and prospective data guiding prevention and management are limited.

Pancreatic manifestations pose diagnostic challenges in distinguishing IBD-related versus medication-induced pancreatitis. Long-term outcomes of type 2 autoimmune pancreatitis and exocrine pancreatic insufficiency remain poorly defined.

Splenic abnormalities, including splenomegaly, granulomas, and functional hyposplenism, have uncertain clinical significance, and oral manifestations demonstrate heterogeneity, with limited mechanistic understanding and evidence-based interventions.

Across all organ systems, most knowledge derives from observational or retrospective studies. There is a paucity of large, prospective trials, highlighting the need for standardized diagnostic criteria, longitudinal data, and multidisciplinary approaches to improve understanding, prevention, and personalized management of DEIMs in IBD [3,11,17,26].

## 7. Future Directions and Research Gaps

Despite advances in understanding extraintestinal manifestations (EIMs) of IBD, important research gaps persist. The pathophysiology of organ-specific complications—including PSC, AIH–PSC overlap, MASLD, and pancreatic or splenic involvement—remains incompletely understood, particularly regarding the gut–liver, gut–pancreas, and oral–gut axes. Standardized diagnostic criteria and validated biomarkers are lacking for several conditions, limiting early detection, risk stratification, and tailored intervention [3,4,73].

Longitudinal, multicenter studies are needed to characterize the natural history of these manifestations, assess long-term outcomes, and evaluate the effectiveness of emerging therapies, including mechanism-based and personalized treatment strategies. Further research should also clarify the impact of IBD therapies on extraintestinal organs, define optimal surveillance protocols, and explore the integration of multidisciplinary, patient-centered care to improve outcomes [3,4,73].

Addressing these gaps will support the development of more precise, personalized, and evidence-based management approaches for patients with IBD, ultimately enhancing both intestinal and systemic health.

## 8. Personalized Management

The development of digestive extraintestinal manifestations (DEIMs) in IBD reflects a complex interplay of intestinal inflammation, genetic susceptibility, microbiome alterations, metabolic factors, prior surgery, and environmental exposures. Because these manifestations cannot be predicted solely by disease subtype, location, or severity, individualized assessment is essential.

DEIMs—including hepatobiliary disorders (PSC, AIH overlap, MASLD, cholelithiasis), pancreatic involvement, splenic abnormalities, and oral lesions—contribute substantially to morbidity and clinical complexity. Effective care requires structured baseline and longitudinal evaluation, incorporating laboratory testing, imaging, and targeted histologic or functional assessment as indicated.

Management should be patient-centered and risk-adapted, balancing optimal IBD control with treatment-related hepatotoxicity, metabolic burden, and comorbidities. Surveillance strategies—such as colonoscopy, hepatobiliary imaging, thrombosis monitoring, and vaccination in hyposplenic patients—should be tailored to each patient’s risk profile. Close multidisciplinary collaboration ensures timely intervention, coordinated organ-specific care, and improved long-term outcomes.

## 9. Conclusions

Digestive extraintestinal manifestations in IBD—including hepatobiliary, pancreatic, splenic, and oral involvement—are common and highly individualized. Their development reflects the complex interplay of intestinal inflammation, genetic predisposition, microbiome composition, and environmental exposures, making prediction based solely on disease location or severity unreliable. Optimal management requires personalized assessment, regular monitoring, risk-adapted surveillance, and multidisciplinary coordination to guide therapy, prevent complications, and improve long-term outcomes for patients with IBD.

## Figures and Tables

**Table 1:** The main digestive extra enteric manifestations of inflammatory bowel disease.

Organ/System	Specific Manifestations	Clinical Notes
**Liver/** **Bile Ducts** [31,36,37,38,39,40,41,42,43,44,45,46,47,48,49,50,51,52,53,54,55,56,57,58]	Primary sclerosing cholangitis (PSC); Autoimmune hepatitis (AIH); Non-alcoholic fatty liver disease (NAFLD); MASLD Drug-induced liver injury (DILI)	Most common digestive EIMs; PSC frequently coexists with ulcerative colitis; predominantly immune-mediated; requires regular biochemical and imaging surveillance
**Gallbladder** [31,59,60]	Cholelithiasis (gallstones)	More frequent in Crohn’s disease, particularly with ileal involvement or resection due to bile salt malabsorption
**Pancreas** [61,62,63,64,65]	Acute pancreatitis; Chronic pancreatitis; Pancreatic exocrine insufficiency	May be idiopathic, autoimmune, or drug-induced (e.g., thiopurines, 5-ASA); important to differentiate from PSC-related pancreatic involvement
**Spleen** [27,31,66]	Splenomegaly; Hypersplenism	Typically, secondary to chronic inflammation, portal hypertension, or hepatobiliary complications
**Oral Cavity** [19,66,67,68,69,70]	Aphthous ulcers; Angular cheilitis; Mucosal inflammation; Granulomatous lesions	May precede intestinal diagnosis; reflects systemic immune dysregulation; more common in Crohn’s disease

## Data Availability

The original contributions presented in this study are included in the article. Further inquiries can be directed to the corresponding author.

## Abbreviations

The following abbreviations are used in this manuscript:

IBD	Inflammatory bowel disease
CD	Crohn’s disease
UC	Ulcerative colitis
IC	Indeterminate colitis
EIMs	Extraintestinal Manifestations
PSC	Primary Sclerosing Cholangitis
DEIMs	Digestive Extraintestinal/Extraenteric Manifestations
HLA	Human Leukocyte Antigen
AIP	Autoimmune Pancreatitis
TNF	Tumor Necrosis Factor
NAFLD	Non-alcoholic fatty liver disease
MRCP	Magnetic Resonance Cholangiopancreatography
ERCP	Endoscopic Retrograde Cholangiopancreatography
SD-PSC	Small-Duct Primary Sclerosing Cholangitis
UCDA	Ursodeoxycholic acid
AIH	Autoimmune hepatitis
ANA	Antinuclear antibodies
SMA	Smooth muscle antibodies
MASLD	Metabolic dysfunction–associated steatotic liver disease
MTX	Methotrexate
5-ASA	5-aminosalicylic acid
DILI	Drug-induced liver injury
PVT	Portal vein thrombosis
PVST	Portal venous system thrombosis
CT	Computed Tomography
MRI	Magnetic Resonance Imaging
EPI	Exocrine pancreatic insufficiency
DMFT	Decayed, Missing, Filled Teeth
